# Serum Uric Acid as an Indicator of Right Ventricular Dysfunction in LVAD Patients: A Preliminary Study

**DOI:** 10.3390/biomedicines12091935

**Published:** 2024-08-23

**Authors:** Tomasz Urbanowicz, Małgorzata Tomaszewska, Anna Olasińska-Wiśniewska, Jędrzej Sikora, Ewa Straburzyńska-Migaj, Jakub Piecek, Maksymilian Białasik-Misiorny, Aleksandra Krasińska-Płachta, Andrzej Tykarski, Marek Jemielity

**Affiliations:** 1 Cardiac Surgery and Transplantology Department, Poznan University of Medical Sciences, 61-107 Poznan, Poland; 2 1st Cardiology Department, Poznan University of Medical Sciences, 61-107 Poznan, Poland; 3 Poznan University of Medical Sciences, 61-107 Poznan, Poland; 4 Department of Ophthalmology, Poznan University of Medical Sciences, 61-107 Poznan, Poland; 5 Department of Hypertensiology, Angiology and Internal Medicine, Poznan University of Medical Sciences, 61-107 Poznan, Poland

**Keywords:** heart failure, LVAD, uric acid, right ventricle, TAPSE

## Abstract

(1) Background: Left ventricular assist devices (LVADs) represent mechanical support in end-stage congestive heart failure and are characterized by satisfactory long-term results. Uric acid (UA) represents one of the early heart failure markers whose usefulness was postulated in clinical practice. (2) Methods: Twenty-nine male patients with a median age of 58 (51–62) years were referred for LVAD implantation due to end-stage congestive heart failure in the mean (SD) New York Heart Association (NYHA) status class 3.3 (0.6). Preoperative and postoperative right ventricular (RV) characteristics were compared with serum uric acid concentration within 12 (8–15) months following the implantation. (3) Results: Significant correlations between postoperative uric acid concentration and right ventricular dimension (r = 0.604, *p* = 0.005), tricuspid annulus plane systolic excursion (TAPSE) (r = −0.496, *p* = 0.022), left ventricular ejection fraction (r = −0.463, *p* = 0.046), and N-terminal pro-B-type natriuretic peptide (NT-pro-BNP) (r = 0.505, *p* = 0.041) were noted. (4) Conclusions: The analysis shows the association between the postoperative RV diameter and TAPSE results in LVAD patients and uric acid concentration. Serum uric acid can be regarded as a possible right ventricular dysfunction marker in LVAD patients.

## 1. Introduction

Heart failure is a current epidemiological burden [1]. The advanced stages of heart failure are characterized by progressive worsening that ultimately may require surgical intervention for life-prolonging therapies such as mechanical support or heart transplantation [2]. A growing number of patients are reported to be candidates for such therapies, though no single test or clinical symptom can identify them [3].

Left ventricular assist devices (LVADs) represent mechanical support in end-stage congestive heart failure and are characterized by satisfactory long-term results [4]. The change in hemodynamics restores cardiac output and reduces left ventricular and pulmonary arterial bed pressures, enabling improved organ perfusion [5]. Despite many advantages and the clinical safety of mechanical support, progressive right ventricular (RV) failure (RVF) has been reported in 5–44% of LVAD patients [6,7] and may impact patients’ survival [8,9]. Though RV function also improves by an immediate reduction in left-sided filling pressures, followed by a decrease in the mitral regurgitation, pulmonary vascular resistance, and reversal of excessive septal muscle shift [6], additional multifactorial mechanisms may hamper the post-LVAD RV performance. This phenomenon may lead to multiorgan failure and represents an ominous sign for patients’ prognosis. 

Moreover, dedicated, effective medical therapy is lacking after this complication occurs. Thus, early diagnosis is of utmost importance. However, the pre-LVAD prediction of possible postoperative RVF in most cases is difficult and inaccurate as the right ventricle is overtly dysfunctional with end-organ involvement [9]. Several parameters and algorithms have been created to stratify RVF risk [10]. However, their predictive value is still limited [10]. Simple and widely available RV assessment tools before LVAD implantation are highly beneficial. 

Uric acid (UA) represents one of the early heart failure markers whose usefulness was postulated in clinical practice [11]. The prevalence of hyperuricemia has been reported in heart failure patients, though recent analyses showed that xanthine oxidase inhibitors may increase mortality in heart failure patients [12]. In animal studies, right ventricle hypertrophy and severe fibrosis were linked to excessive xanthine and uric acid accumulation [13]. 

This study aimed to assess the clinical usefulness of UA concentration measurements to differentiate LVAD patients at higher risk of post-LVAD RV dysfunction (RVD).

## 2. Materials and Methods

In this single-center, prospective study, consecutive patients presenting with end-stage heart failure referred for LVAD implantation with HeartMate3 (Abbott Medical, Pleasanton, CA, USA) were enrolled. Clinical and demographic data were collected. Symptoms of heart failure were evaluated based on New York Heart Association (NYHA) functional classification. Postprocedural presentation and complications were recorded. 

The RVD after LVAD implantation was assessed by echocardiography. Transthoracic echocardiography (TTE) was performed before LVAD implantation, after the procedure, and at the follow-up by experienced echocardiographers according to the same protocol. Left ventricular (LV) and RV diameters, left ventricular ejection fraction (LVEF), and valvular insufficiencies were evaluated before the procedure. RV diameters were assessed in the parasternal long-axis and apical 4-chamber views. Tricuspid annular plane systolic excursion (TAPSE) was measured using M-mode in the apical four-chamber view. The preoperative RV parameters were compared to the results obtained in the follow-up. The postoperative RV performance characteristics were compared with preoperative and postoperative laboratory parameters to estimate their possible pathophysiological and predictive role in post-LVAD RVD. 

Right heart catheterization (RHC) was performed preoperatively to assess the cardiac index (CI), cardiac output (CO), pulmonary artery pressure (PAP), pulmonary capillary wedge pressure (PCWP), right atrial pressure, systemic vascular resistance, and pulmonary vascular resistance. 

Blood samples were collected before and after LVAD implantation for blood morphology and biochemical analysis, including N-terminal pro-B-type natriuretic peptide (NT-pro-BNP) and UA concentration. UA normal ranges were established at Sysmex Europe GmbH, Norderstedt, Germany.

The exclusion criteria included the following:

- Preoperative hyperuricemia, either primary or secondary to hypothyroidism;
- End-stage kidney failure;
- Iron supplementation;
- Alcoholic beverage consumption.

None of the patients were pharmacologically treated due to hyperuricemia. 

Patients were divided into two subgroups based on the RV diameter changes in the follow-up: patients with stable or decreased RV dimension and patients with RV dilatation. 

### Statistical Analysis

Continuous variables were reported as means with standard deviations or medians and interquartile ranges (Q1–Q3), depending on the normal distribution results. Categorical data were presented as numbers and percentages. Where applicable, numerical variables were compared using the Mann–Whitney test or repeated measures ANOVA. Fisher’s exact test analyzed categorical data. Spearman correlation analysis was used. Statistical analysis was performed using JASP statistical software (JASP Team; 2023. Version-0.18.1). *p* < 0.05 was considered statistically significant.

## 3. Results

Twenty-nine male patients with a median (Q1–3) age of 58 (51–62) years were referred for LVAD implantation due to end-stage heart failure in the mean (SD) NYHA status class 3.3 (0.6). There were 15 (52%) and 14 (48%) patients diagnosed with ischemic and dilated cardiomyopathy, respectively. Patients were characterized by a median (Q1–3) body mass index (BMI) of 27.5 (25.1–28.7). Atrial fibrillation was diagnosed in 16 (55%) patients and arterial hypertension in 10 (35%) patients, and 8 (28%) reported diabetes mellitus type 2. 

Preoperatively, 24 patients were amin pressor-dependent, and 1 patient required intra-aortic balloon pump (IABP) support. Levosimendan was administered routinely to all the patients one day before the procedure. The preoperative values of right heart catheterization revealed cardiac index (CI) median (Q1–3) values of 1.99 (1.82–2.13) L/min/m^2^, followed by pulmonary artery pressure (PA) and pulmonary vascular resistance (PVR) values of 240 (120–336) and 26 (19–47) mmHg, respectively.

All patients underwent HeartMate 3 (Abbott Medical, Pleasanton, CA, USA) implantation through median sternotomy with cardiopulmonary support. There were no perioperative deaths in the presented group. The median (Q1–3) hospitalization and follow-up time were 31 (24–36) days and 12 (8–15) months, respectively. Postoperatively, six (21%) patients required prolonged mechanical ventilation, and pleurecentesis was performed on seven (24%) patients. Transient ischemic attack was diagnosed in two (7%) patients. Following surgery, all patients were hemodynamically stable and presented in NYHA I. None of them either presented RV failure at the stage requiring additional mechanical RV support or required pharmacotherapy due to preoperative hyperuricemia. A significant clinical improvement followed by an NT-pro-BNP decrease was observed (*p* = 0.006). Preoperatively, five (17%) presented with increased uric acid serum concentration related to severe congestive heart failure. Postoperatively, an increased uric acid concentration was noted in 17 (31%) patients, indicating a statistical difference (*p* = 0.003). The preoperative and follow-up laboratory results were compared and are presented in Table 1. 

The preoperative and postoperative echocardiographic parameters were evaluated (Table 2). We focused on the preoperative values of right ventricular diameter (RV) that were 31 (29–37) mm and TAPSE reaching the value of 14.5 (14.0–16.8 mm). The postoperative RV dimensions and TAPSE values were 32.5 (28.5–38.8) mm and 14 (13–15) mm, respectively. There were no significant differences between the preoperative and postoperative values of right ventricular diameter (*p* = 0.567) nor TAPSE (*p* = 0.140). 

In the follow-up, 22 patients presented with stable or increased RV diameter parameters (group 1), while in 7 (24%) patients, RV diameter distension was noted (group 2). There were no significant differences between the groups regarding age (*p* = 1.000), BMI (*p* = 0.861), or primary diagnosis (*p* = 0.843). We did not notice significant differences regarding co-morbidities, including arterial hypertension (*p* = 0.242), diabetes (*p* = 0.105), or atrial fibrillation (*p* = 0.552). Kidney function tests presented by the glomerular filtration rate were indifferent (*p* = 0.895), as were preoperative uric acid serum concentrations (*p* = 0.533). 

**Figure 1:**
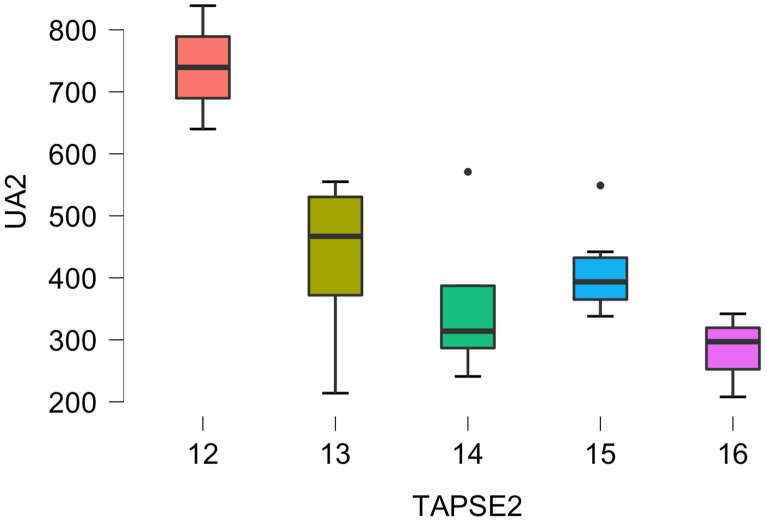
The post-implantation uric acid serum concentration (UA2) depends on postoperative TAPSE (TAPSE-2) in LVAD patients.

Patients presenting right ventricular dilatation in follow-up were characterized by significant differences in the median (Q1–3) TAPSE (14.5 (13.3–15.8) mm vs. 13.0 (12.5–14.0) mm, *p* = 0.050). The relation between postoperative TAPSE and UA concentrations is presented in Figure 1.

Laboratory and LVAD function parameters were analyzed in both subgroups (Table 3). A statistically significant difference regarding the serum concentration of uric acid (*p* = 0.002) next to NT-pro-BNP (*p* = 0.006) was noted.

**Figure 2:**
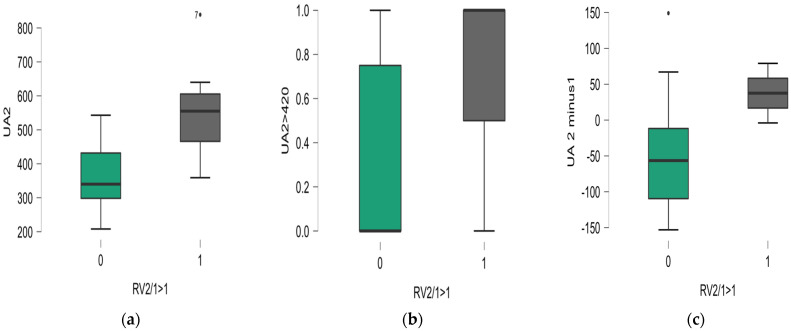
Differences between uric acid serum concentration (**a**), number of hyperuricemic patients (**b**), and serum uremic acid pre-LVAD and post-LVAD concentration differences (**c**) related to post-to-preoperative RV diameter ratio.

There was a significant difference regarding UA serum concentration in LVAD patients associated with RV performance, as presented in Figure 2a–c.

### 3.1. Correlations

A significant correlation was found between postoperative serum uric acid concentration and right ventricular postoperative diameter (RV2) (r = 0.604, *p* = 0.005). The association was also confirmed when the postoperative-to-preoperative RV diameter ratio (RV2/RV1 ratio) was correlated with uric acid (r = 0.612, *p* = 0.014). The presented relations were not observed between preoperative measurements (UA1 vs. RV1: r = 0.523, *p* = 0.229). A significant negative correlation between postoperative TAPSE and uric acid concentration was noted (r = −0.496, *p* = 0.022).

No significant relations were noted between preoperative uric acid and preoperative left ventricular diameter (r = 0.214, *p* = 0.662) nor postoperative serum uric acid and left ventricular diameter (r = 0.366, *p* = 0.113).

Moreover, there was no correlation between preoperative UA and LVEF1 (r = 0.256, *p* = 0.574) nor between respective postoperative parameters. In follow-up observations, a significant negative correlation between left ventricular ejection fraction and uric acid was observed (r = −0.463, *p* = 0.046). The postoperative values of NT-pro-BNP correlated with uric acid (r = 0.505, *p* = 0.041).

The possible relation between postoperative uric acid concentration and pump flow (r = 0.499, *p* = 0.082) and pump speed (r = −0.185, *p* = 0.478) did not reach statistical significance.

### 3.2. Receiver Operator Curve (ROC)

The predictive value for right ventricular deterioration (RV2/RV1) following LVAD implantation was measured by ROC analysis. The postoperative uric acid serum concentration revealed an area under the curve of 0.898, yielding a sensitivity of 71.4% and a specificity of 92.2%. The possible predictive value of UA serum concentration difference between postoperative and preoperative values was not confirmed.

## 4. Discussion

The results of our study point out the strong association between serum UA concentration and the dilation of the RV diameter after LVAD implantation. The relation between serum uric acid and right ventricular dimension was not observed preoperatively. More interestingly, the presented link was noticed postoperatively for all LVAD patients, and statistical differences between uric acid and RV diameter progression were observed.

This is the first study assessing the relationship between UA and RV performance in patients after LVAD implantation. UA induces oxidative stress and leads through inflammatory processes to endothelial dysfunction [14]. On the contrary, a decrease in serum acid was found to be significant for improving endothelial function [15]. Decreased serum uric acid can improve endothelial function [15,16]. In turn, endothelial dysfunction correlates with pulmonary hypertension [17]. In Du et al.’s study, UA was revealed as an independent risk factor of adverse outcomes in left ventricular failure with pulmonary hypertension [18]. The relationship between hyperuricemia and left and right ventricular function was postulated in atrial fibrillation, as presented by Jordhani et al.’s analysis [19]. This biologically active substance is claimed to participate in human heart failure (HF) progression [20].

LVAD therapy is a compelling, long-term mechanical circulatory support that replaces left ventricular function but requires the right ventricle (RV) to maintain its function [6]. The follow-up NT pro-BNP serum concentration drop in our analysis confirmed the clinical improvement following LVAD implantation. The risk for progressive RV dysfunction following LVAD implantation is reported in over 20% of patients [21].

RV dysfunction begins with initial dyssynchrony, ventricular dilation, and increased wall tension and oxygen demand, followed by decreased RV contractility [22]. The implantation of an LVAD that replaces the left-sided heart function may induce additional stress on the right ventricle [9], which, according to our results, may be described by uric acid concentration. Our study shows the relationship between serum uric acid and RV characteristics in LVAD patients. It shows statistically significant differences in the subgroup presenting with RV deterioration, indicating more profound oxygenation stress activation while the RV proceeds to impairment. We may conclude that the serum concentration of uric acid in LVAD patients can be regarded as an early marker of RV deterioration and suggest that RV hemodynamics induce oxidative stress processes.

UA exhibits proinflammatory properties and has been reported as a marker of impaired oxidative metabolism, inflammatory cytokine activation, and impaired vascular function in chronic heart failure [23]. It is capable of inducing cell death [24]. UA reflects the degree of circulating xanthine oxidase (XO) activity, a source of oxygen free radicals, and oxidative stress-induced cardiac injury [25]. Remarkably, inflammation and oxidative stress are associated with cardiovascular risk and worse outcomes [26]. Moreover, kidney dysfunction and diuretics may contribute to UA concentration.

A UA increase reflects the interplay between metabolic processes and cardiovascular dysfunction [27]. A cohort study by Wu et al. with 2749 participants identified hyperuricemia as a predictor of the development of chronic heart failure in patients over 65 years [28]. Hyperuricemia and a high UA-to-creatinine ratio were associated with an increased risk of mortality in heart failure [17,18,19,20]. Piani et al. showed that the UA-to-GFR ratio correlated with the composite outcome of death or re-hospitalization for acute heart failure [29]. Miao et al. explored the correlation between UA and prognosis in patients with chronic heart failure after coronary revascularization. UA, together with LVEF, happened to be better predictors of poorer outcomes than NT pro-BNP or LV diameter [30]. Our study did not show the association between NT pro-BNP and UA concentration and LVEF, pre- and postoperatively; however, it pointed out the significance of UA measurements in determining the worsening of RV function.

Our results may help to point out the subgroup of patients who are at risk for RV deterioration, which is a morbid complication after LVAD implantation. The significance of our study is based on a simple, easily accessible parameter that could be considered as a useful predictor in clinical practice.

All patients included in this study presented several similarities in terms of heart failure advancement with severely decreased LVEF. We estimated that this homogeneity may explain the lack of preprocedural correlations between NT pro-BNP, UA, and echocardiographic parameters. At the same time, the change in RV diameter was considered an adverse outcome and was associated with UA concentration. RV performance was acceptable before implantation. UA differentiated patients in whom RV began to deteriorate after the procedure. Amin et al. [31] reported elevated uric acid levels in patients with systolic heart failure as associated with impaired clinical and hemodynamic profile. Our study underlined the significance of UA in RV assessment in advanced left ventricular heart failure with LVAD support.

The early differentiation of patients at a higher risk of RV failure is crucial after LVAD implantation [9]. Our previous report assessment of neutrophil to extracellular traps (NET) was revealed as significant for RV dilatation [32]. However, NET represents a more sophisticated laboratory method. We have now shown that UA is a valuable and simple laboratory marker of RV dilation, the early stage of RV deterioration. According to our study results, increased UA will prompt clinicians to perform control echocardiography and optionally intensify therapy to avoid progression to RV failure.

## 5. Conclusions

This analysis shows the association between the postoperative RV diameter and TAPSE results in LVAD patients and uric acid concentration. Serum uric acid can be considered a possible right ventricular dysfunction marker in LVAD patients. Therefore, serum uric acid could be collected in heart failure patients, particularly before and consecutively after LVAD implantation, to reveal the potential risk of RV dysfunction. Increasing serum uric acid concentration could raise clinical awareness and suggest the implementation of prompt imaging techniques to refine the diagnosis. Further studies are required to validate these preliminary findings and fully understand their clinical implications.

## Figures and Tables

**Table 1:** Preoperative and follow-up laboratory results.

Parameters (Median (Q1–Q3))	Preoperative *n* = 29	Follow-Up *n* = 29	*p*
Peripheral blood analysis:			
WBC (K/μL)	7.93 (6.89–8.54)	7.99 (6.76–9.60)	0.747
Neutrophile (K/μL)	5.52 (4.59–6.50)	5.31 (4.33–6.31)	0.603
Lymphocyte (K/μL)	1.32 (0.95–1.90)	1.29 (1.09–1.70)	0.237
Monocyte (K/μL)	0.52 (0.43–0.58)	0.51 (0.43–0.63)	0.227
Hemoglobin (mmol/L)	8.80 (7.50–9.46)	8.60 (7.35–8.80)	0.438
Hematocrit (%)	43 (36–45)	41 (35–44)	0.300
Platelets (K/μL)	197 (157–254)	198 (167–251)	0.705
MPV (fL)	8.65 (8.43–9.50)	8.30 (8.00–9.13)	0.722
MCV (fL)	91.5 (89.0–95.0)	88 (81.8–90.4)	0.023
MCHC (mmol/L)	20.79 (20.54–20.96)	20.56 (20.21–21.02)	1.000
RDW (%)	15.0 (14.3–15.6)	15.3 (14.6–16.6)	0.925
Kidney tests:			
Creatinine (mmol/L)	115 (86–137)	109 (87–136)	0.227
GFR (mL/min)	71 (58–89.5)	61 (47.5–73.5)	0.374
UA (μmol/L)	396.5 (295.8–470.8)	383 (326–543)	0.492
UA > 420 μmol/L	5 (17)	9 (31)	0.003 *
LDH (U/L)	237 (218–290)	221 (201–246)	0.213
NT pro-BNP (pg/mL)	2873 (1789–4372)	916 (432–1821)	0.006 *

Abbreviations: GFR—glomerular filtration rate, LDH—lactate dehydrogenase, MCV—mean corpuscular volume, MCHC—mean corpuscular hemoglobin concentration, MPV—mean platelet volume, NT pro-BNP—N-terminal pro-B-type natriuretic peptide, RDW—red cell distribution width, UA—uric acid, WBC—white blood cell count. * statistically significant.

**Table 2:** Preoperative and follow-up echocardiographic parameters.

Echocardiography Data	Preoperative *n* = 29	Follow-Up *n* = 29	*p*
LV mm (median (Q1–Q3))	65 (60–75)	63.5 (57–68.8)	0.147
RV mm (median (Q1–Q3))	31 (29–37)	32.5 (28.5–38.8)	0.567
RV2/1 (median (Q1–Q3))	NA	0.93 (0.91–1.08)	NA
RV2/RV1 > 1 (n, (%))	NA	7 (24)	NA
RV2–RV1 difference (mm) (median (Q1–Q3))	NA	−1.5 (−3.3–19.00)	NA
TAPSE mm (median (Q1–Q3))	14.5 (14.0–15.5)	15.0 (14.0–16.0)	0.140
TAPSE2–TAPSE1 difference (mm) (median (Q1–Q3))	NA	0 (−5–13)	NA
LA mm (median (Q1–Q3))	48 (44–52)	49 (48–51.8)	0.615
IVs mm (median (Q1–Q3))	10 (9–11)	10 (10–11)	0.380
IM grade (median (Q1–Q3))	2.5 (2.1–3.1)	2.3 (2.1–2.7)	0.643
LVEF (%) (median (Q1–Q3))	20 (15–25)	20 (15–20)	0.633

Abbreviations: IM—mitral regurgitation, IVs—intraventricular septum, LA—left atrium, LV—left ventricular diameter, LVEF—left ventricular ejection fraction, NA—not applicable, RV—right ventricular diameter, RV2/1—postoperative/preoperative right ventricular ratio, TAPSE—tricuspid annular plane systolic excursion.

**Table 3:** A comparison of laboratory and pump parameters between two subgroups of LVAD patients.

Parameters (Median (Q1–Q3))	Stable or Decreased RV Dimension (*n* = 22)	RV Dilatation (*n* = 7)	*p*
Peripheral blood analysis:			
WBC (K/μL)	7.78 (6.06–9.02)	7.97 (7.81–8.15)	0.630
Neutrophile (K/μL)	6.02 (4.57–6.83)	5.02 (4.94–6.20)	0.447
Lymphocyte (K/μL)	1.21 (0.93–1.87)	1.63 (1.52–1.91)	0.944
Monocyte (K/μL)	0.46 (0.42–0.56)	0.58 (0.57–0.66)	0.078
Hemoglobin (mmol/L)	8.90 (6.70–9.50)	8.70 (6.70–9.50)	0.620
Hematocrit (%)	43 (35–45)	44 (38–45)	0.479
Platelets (K/μL)	195 (156–257)	200 (197–244)	0.657
MPV (fL)	9.40 (8.40–9.70)	8.50 (8.30–8.70)	0.972
MCV (fL)	93 (89–96)	90 (89–94)	0.916
MCHC (mmol/L)	20.94 (20.51–20.99)	20.73 (20.62–20.76)	0.573
RDW (%)	15.0 (14.3–15.6)	15.0 (14.6–15.2)	0.646
Other:			
Creatinine (mmol/L)	113 (90–135)	125 (84–125)	0.057
GFR (mL/min)	70.5 (58.0–88.5)	67.0 (55.5–78.5)	0.218
LDH (μmol/L)	262.5 (229.3–299.8)	219.5 (202.8–246.8)	0.452
UA (μmol/L)	396.5 (288.3–454.3)	428.5 (366.3–491.8)	0.002 *
UA difference (2 − 1)	−57 (−110–−12)	38 (17–58)	0.041
NT pro BNP (pg/mL)	759 (484–1906)	1072 (414–1332)	0.775
LVAD parameters:			
Flow (L/min)	4.5 (4.0–4.7)	4.6 (4.4–6.1)	0.177
Speed (RPM)	5.200 (5050–5225)	5200 (5000–5300)	0.882

Abbreviations: GFR—glomerular filtration rate, LDH—lactate dehydrogenase, MCV—mean corpuscular volume, MCHC—mean corpuscular hemoglobin concentration, MPV—mean platelet volume, NT pro-BNP—N-terminal pro-B-type natriuretic peptide, RDW—red cell distribution width, RPM—revolutions per minute, UA—uric acid, WBC—white blood cell count, * statistically significant.

## Data Availability

The data supporting the reported results can be obtained after a reasonable request from the corresponding authors via email contact within three years following publication.
